# Structural Basis of Specific Glucoimidazole and Mannoimidazole Binding by Os3BGlu7

**DOI:** 10.3390/biom10060907

**Published:** 2020-06-15

**Authors:** Bodee Nutho, Salila Pengthaisong, Anupong Tankrathok, Vannajan Sanghiran Lee, James R. Ketudat Cairns, Thanyada Rungrotmongkol, Supot Hannongbua

**Affiliations:** 1 Center of Excellence in Computational Chemistry (CECC), Department of Chemistry, Faculty of Science, Chulalongkorn University, Bangkok 10330, Thailand; b.nutho@gmail.com; 2 School of Chemistry, Institute of Science, Suranaree University of Technology, Nakhon Ratchasima 30000, Thailand; mai.salila@yahoo.com (S.P.); tankrathok@yahoo.com (A.T.); 3 Center for Biomolecular Structure, Function and Application, Suranaree University of Technology, Nakhon Ratchasima 30000, Thailand; 4 Department of Chemistry, Faculty of Science, University of Malaya, Kuala Lumpur 50603, Malaysia; vannajan@um.edu.my; 5 Biocatalyst and Environmental Biotechnology Research Unit, Department of Biochemistry, Faculty of Science, Chulalongkorn University, Bangkok 10330, Thailand; 6 Program in Bioinformatics and Computational Biology, Graduate School, Chulalongkorn University, Bangkok 10330, Thailand

**Keywords:** transition state mimics, β-glycosidase, X-ray crystallography, MD simulation, REMD

## Abstract

β-Glucosidases and β-mannosidases hydrolyze substrates that differ only in the epimer of the nonreducing terminal sugar moiety, but most such enzymes show a strong preference for one activity or the other. Rice Os3BGlu7 and Os7BGlu26 β-glycosidases show a less strong preference, but Os3BGlu7 and Os7BGlu26 prefer glucosides and mannosides, respectively. Previous studies of crystal structures with glucoimidazole (GIm) and mannoimidazole (MIm) complexes and metadynamic simulations suggested that Os7BGlu26 hydrolyzes mannosides via the *B*_2,5_ transition state (TS) conformation preferred for mannosides and glucosides via their preferred ^4^*H*_3_/^4^*E* TS conformation. However, MIm is weakly bound by both enzymes. In the present study, we found that MIm was not bound in the active site of crystallized Os3BGlu7, but GIm was tightly bound in the −1 subsite in a ^4^*H*_3_/^4^*E* conformation via hydrogen bonds with the surrounding residues. One-microsecond molecular dynamics simulations showed that GIm was stably bound in the Os3BGlu7 active site and the glycone-binding site with little distortion. In contrast, MIm initialized in the *B*_2,5_ conformation rapidly relaxed to a *E*_3_/^4^*H*_3_ conformation and moved out into a position in the entrance of the active site, where it bound more stably despite making fewer interactions. The lack of MIm binding in the glycone site in protein crystals and simulations implies that the energy required to distort MIm to the *B*_2,5_ conformation for optimal active site residue interactions is sufficient to offset the energy of those interactions in Os3BGlu7. This balance between distortion and binding energy may also provide a rationale for glucosidase versus mannosidase specificity in plant β-glycosidases.

## 1. Introduction

The largest source of renewable biomass on earth is plant-derived carbohydrate, including cellulose and other β-glucans, as well as xylans, mannans and mixed polysaccharides. The breakdown of these polysaccharides plays an important role in biomass conversion and biofuel production [1]. The abundant β-glucan biomass serves as a nutritional source for various organisms, particularly bacteria and fungi. Plants also need to recycle their cell walls for cell growth and metabolism; thus, they have independently developed their own set of enzymes for β-glucan breakdown. The glycosidases involved in the degradation of β-glucan comprise endoglucanases, exoglucanases and β-glucosidases [2]. These enzymes have been categorized in protein-sequence-similarity-based families in the Carbohydrate-Active enZYme (CAZY) database (www.cazy.org) [3]. CAZymes play a variety of functions in all domains of living organisms, with the greatest expansion of these roles in plants, which have the widest range of carbohydrate-containing molecules.

β-Glucosidases (β-d-glucopyranoside glucohydrolases, E.C. 3.2.1.21) are enzymes that hydrolyze β-*O*-linked glycosidic linkages of glycosides and oligosaccharides to liberate nonreducing terminal d-glucosyl residues [4]. These enzymes have been classified into glycoside hydrolase (GH) families GH1, GH2, GH3, GH5, GH9, GH30, GH39, and GH116 [4,5,6]. Of these, GH1 contains the largest number of well-characterized and structurally defined β-glucosidases [3,7].

β-Glucosidases show a wide range of substrate recognition and specificity to the enzyme, from enzymes that can hydrolyze a single substrate to enzymes that act on a large number of glycosyl or non-glycosyl aglycone moieties [8,9,10,11]. Most β-glucosidases are thought to catalyze hydrolysis through a two-step retaining mechanism involving two catalytic carboxylate residues (two glutamate residues for GH1 enzymes), one of which serves as a general acid/base catalyst and another as a nucleophile [12,13]. In the glycosylation step, the catalytic acid protonates the aglycon leaving group and then the nucleophilic residue attacks the anomeric carbon to displace the aglycon and consequently form a covalent glycosyl-enzyme intermediate. In the deglycosylation step, a water molecule or another nucleophile is deprotonated by the catalytic acid/base and attacks the anomeric carbon to release β-d-glucose from the enzyme. Both steps are thought to proceed via the formation of oxocarbenium cation-like transitions states (TS). These TS are stabilized by a partial double bond between the anomeric carbon (C1) and the endocyclic oxygen (O5), which requires that the atoms C5–O5–C1–C2 are coplanar, or nearly so, at the TS [14]. TS conformations that meet this requirement consist of two boats (*B*_2,5_ and ^2,5^*B*), two half chairs (^4^*H*_3_ and ^3^*H*_4_), and the related envelopes (^4^*E*, *E*_4_, ^3^*E* and *E*_3_) [15].

**Figure 1:**
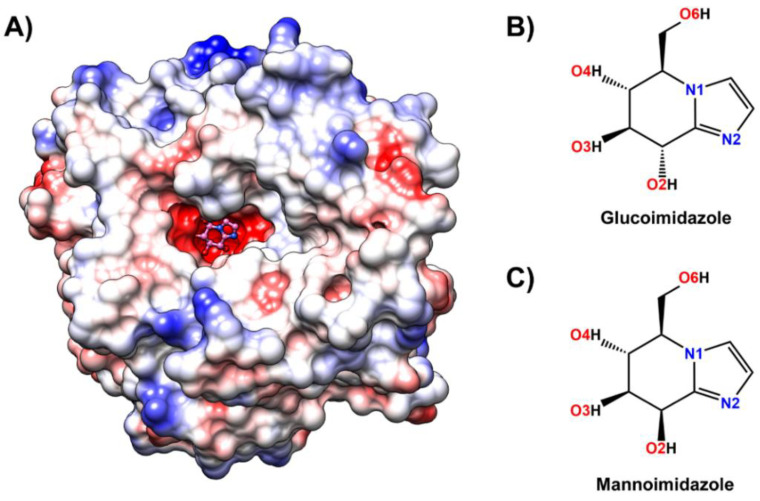
(**A**) Three-dimensional structure of glucoimidazole (pink molecule with ball and stick representation) bound to the active site of Os3BGlu7 β-glucosidase solved in this study (Protein Data Bank (PDB) ID: 7BZM), where the positive and negative charge accumulation are represented by the surface charge ranging from blue to red, respectively. Chemical structure of (**B**) glucoimidazole and (**C**) mannoimidazole. The atomic labels used for further analysis are also given.

Currently, the X-ray crystal structures of GH1 β-glucosidases from bacteria [16], fungi [17], and plants (e.g., rice, maize and wheat) have been extensively studied [8,18,19,20]. Among all the plant enzymes, one of the most well-studied enzymes is rice (*Oryza sativa* L.) Os3BGlu7 β-glucosidase (Figure 1A), originally designated as rice BGlu1, which was identified as a highly expressed iso-enzyme in rice flower and geminating shoot [18,19,21,22]. Rice Os3BGlu7 acts as an exo-β-glucosidase on β-1,3- and β-1,4-linked gluco-oligosaccharides and also exhibits transglucosylation activity toward these substrates [21,22]. Kinetic subsite analysis of cellooligosaccharide hydrolysis suggested that rice Os3BGlu7 had at least six subsites for binding of β-1,4-linked d-glucosyl residues [23].

Rice Os3BGlu7 and a set of closely related plant enzymes including rice Os3BGlu8 and Os7BGlu26 and barley HvBII were found to have both β-glucosidase and β-mannosidase activities [22,24,25,26]. Os3BGlu7 hydrolyzes 4-nitrophenyl (4NP) β-d-glucosides with higher efficiency (i.e., *k*_cat_/*K*_M_ is 34-fold higher for 4NP β-d-glucoside than for 4NP β-d-mannoside), while Os7BGlu26 and HvBII show the opposite preference. In general, β-glucosidases are thought to catalyze the first step of β-glucoside hydrolysis via a ^1^*S*_3_ to ^4^*H*_3_/^4^*E* to ^4^*C*_1_ glycone conformational itinerary, while β-mannosidases use a ^1^*S*_5_ to *B*_2,5_ to ^O^*S*_5_ itinerary [15]. Given the difference observed in the hydrolysis of the glucoside and mannoside substrates, it is of great interest to understand the basis of glycone specificity in GH1 enzymes that have dual β-d-glucosidase and β-d-mannosidase functions.

One powerful strategy to assign conformational itineraries or describe how molecules change shape along a reaction coordinate is the use of TS analogue inhibitors [27]. In fact, the investigation of TS conformation is useful for predicting conformational itineraries (from reactant to product state via the TS) based on the principle that the reaction pathway with the least nuclear motion, i.e., the least change in atomic position and electronic configuration will be favored [28]. Glucoimidazole (GIm) and mannoimidazole (MIm) inhibitors (Figure 1B,C) are qualitatively good models of a pyranosyl oxocarbenium ion-like TS for rice Os7BGlu26 β-mannosidase and similarly showed inhibition of Os3BGlu7 [29]. Os3BGlu7 had 4420-fold higher affinity (lower competitive inhibition constant) for GIm than for MIm, corresponding to a ~5 kcal/mol difference in binding free energy [29]. However, Os7BGlu26 β-mannosidase also showed ~5 kcal/mol more favorable binding energy for GIm than MIm, despite binding MIm in a TS-like conformation and showing a preference for mannoside over glucoside for hydrolysis.

To gain more insight into the basis for β-glucosidase versus β-mannosidase specificity of Os3BGlu7, we compared GIm and MIm binding. Attempts to solve the structure of MIm in the active site of Os3BGlu7 resulted in the structure with GIm, apparently a minor contaminant in the MIm preparation. So, the protein−ligand interactions and binding affinity of GIm and MIm toward Os3BGlu7 β-glucosidase were investigated by means of molecular dynamics (MD) simulations and several binding free energy calculations to show that the stable binding was found in the low energy conformation of the inhibitors, but only GIm bound productively in the substrate glycone position.

## 2. Materials and Methods

### 2.1. Protein Expression, Purification, Crystallization and Structure Determination

Rice Os3BGlu7 was expressed in *Escherichia coli* strain Origami (DE3) and purified by immobilized metal affinity chromatography (IMAC), enterokinase digestion and IMAC, as previously described [19,21]. MIm was generously provided by Spencer J. Williams and was synthesized, as previously described [30]. The Os3BGlu7 was crystallized by hanging drop vapor diffusion with microseeding, optimizing polyethylene glycol mono-methyl ether (PEG MME) 5000 concentration in the range of 20–26%, (NH_4_)_2_SO_4_ from 0.16–0.26 M, and protein from 1–5 mg/mL in 0.1 M MES, pH 6.7, at 288 K, as previously described [19]. Prior to flash cooling in liquid nitrogen, the crystals were soaked with 10 mM mannoimidazole (MIm) in cryo solution for 5 min. Diffraction data were collected at the Spring-8 synchrotron beamline BL44XL with 0.9000 Å X-ray radiation on a MX-300HE detector (Rayonix). The data were processed and scaled with the HKL2000 suite [31]. The Os3BGlu7 complex structure was solved by the rigid body refinement of the native Os3BGlu7 structure (Protein Data Bank (PDB) code 2RGL). The refinement was executed with REFMAC5 with tight noncrystallographic symmetry restraints and model building with Coot [32]. The glucosyl residue were built into the electron densities in the shapes that fit the densities best and refined. The refined sugar residue coordinates were assigned their final conformation designation according to their Cremer–Pople parameters [33], calculated by the Cremer–Pople parameter calculator of Prof. Shinya Fushinobu (University of Tokyo, http://enzyme13.bt.a.u-tokyo.ac.jp/CP/). The final models were analyzed with PROCHECK [34] and validated on the PDB website.

### 2.2. System Preparation for Molecular Modeling

The starting coordinates of the Os3BGlu7 β-glucosidase in complex with GIm were taken from our X-ray structure, as shown in Figure 1A. For constructing the Os3BGlu7−MIm complex, the coordinates of the GIm complex were overlaid with those of the Os7BGlu26 β-mannosidase crystalized with MIm (PDB accession code 4RE2) [29]. The initial conformations of the Os3BGlu7−GIm and Os3BGlu7−MIm structures used for MD simulation are illustrated in Figure S1, Supplementary Information (SI). To prepare the apo protein, the GIm was deleted from the complex structure. The ionizable residues were assigned at pH 7.0 using the H++ web-prediction of protonation (http://biophysics.cs.vt.edu/H++) [35], except that the catalytic acid E176 was modeled as the protonated form (GLH type in AMBER format) owing to mechanistic consideration [36]. Moreover, all histidine residue protons were selected based on their possible hydrogen bond network with the surrounding residues. The partial atomic charges of glycoside inhibitors were calculated with the restrained electrostatic potential (RESP) method at the HF/6-31G(d) level of theory using the Gaussian09 program [37]. The ff14SB AMBER force field [38] was applied for the protein, while the generalized Amber force field version 2 (GAFF2) [39] was used to treat the inhibitor. Each system was embedded in the TIP3P [40] water box with the minimum buffer of 10 Å around the protein and neutralized with 11 chloride ions [41]. A system was composed of 7446 protein atoms and roughly 15,000 water molecules enclosed in a box with dimensions of 76 Å × 87 Å × 87 Å. To remove bad atomic contacts, the added hydrogen atoms and solvent molecules were subjected to 1000 steps of steepest descent (SD) energy minimization, followed by 2000 steps of conjugated gradient (CG) method, while the rest of the atoms were restrained. Afterward, the protein and inhibitor were minimized by SD (1000 iterations) and CG (2000 iterations) methods with the restrained solvent. In the final step, the whole system was fully minimized by the same minimization protocol.

### 2.3. Molecular Dynamics Simulations

Molecular Dynamics (MD) simulations of the Os3BGlu7−inhibitor complexes were performed using the AMBER16 software package coupled with the SANDER and PMEMD modules [42] under periodic boundary conditions. Once the system was energy-minimized as described above, the MD protocol firstly consisted of a heating step (from 10 to 300 K) for 100 ps with positional restraints of 20.0 kcal/(mol·Å^2^) applied to the protein Cα atoms. Afterward, the system was equilibrated over 5000 ps, which was subdivided into five steps of restrained MD equilibrations at a constant temperature of 300 K with decreased positional restraints of 20, 15, 10, 5 and 2.5 kcal/(mol·Å^2^) every 1000 ps. Finally, the entire system was run under isothermal-isobaric (*NPT*) conditions (*P* = 1 atm and *T* = 300 K) reaching 1 µs without any positional restraints. Along the MD simulation, a cut-off distance of 10 Å was applied for non-bonded interactions. All covalent bonds containing hydrogen were constrained using the SHAKE algorithm [43], and the long-range electrostatic interactions were treated using the Particle Mesh Ewald method [44]. The Langevin thermostat [45] and the Berendsen barostat [46] were used to control temperature and pressure, respectively. A 2-fs simulation time step was used throughout the MD simulation. The MD trajectories were analyzed over the time period of 600–1000 ns, after equilibration phase was certainly reached. The analysis of the MD trajectories was performed using the CPPTRAJ utility [47] of AmberTools16. The estimation of binding free energies for the rice Os3BGlu7−inhibitor complexes was calculated by Molecular Mechanics/Poisson–Boltzmann (generalized Born) Surface Area (MM/PB(GB)SA) and Quantum Mechanics/generalized Born Surface Area (QM/GBSA) methods using the MMPBSA.py [48] module of AMBER16. In the latter approach, the inhibitor was only included in the QM region and treated by the self-consistent charge density functional tight binding (SCC-DFTB) semiempirical method, while the rest of atoms were described at the MM level using the ff14SB AMBER force field. The same sets of MD snapshots were also used to predict binding free energy on the basis of the solvated interaction energy (SIE) method [49,50]. Apart from the aforementioned approaches, the WaterSwap method from the Sire package [51] was applied to calculate the binding free energy using an explicit water model by swapping the ligand dimensions with an equal shape and volume of explicit water molecules within the protein-binding site [52]. From a 1 µs trajectory for Os3BGlu7–GIm and Os3BGlu7–MIm complexes, 5 representative snapshots were taken every 100 ns between 600−1000 ns and employed for WaterSwap calculations. Algorithms for energy calculations were evaluated using four different statistical techniques: thermodynamic integration (TI), quadrature-based integration of TI, free energy perturbation (FEP) and the Bennett’s acceptance ratio (BAR) method. The degree of convergence of the calculated binding free energy was assessed by taking into account the agreement value between these four estimates, where, ideally, the deviation should be roughly equal [52,53,54,55].

### 2.4. Replica Exchange Molecular Dynamics of the Unbound Glycoside Inhibitors

In this work, replica exchange molecular dynamics (REMD) simulations were applied to study the sugar ring conformation of GIm and MIm in a free form in an explicit water solvation model. The system preparations and REMD simulations were carried out using the AMBER16 software package. The initial structures of the free GIm and MIm were extracted from the co-crystal structures of the Os3BGlu7−GIm and Os7BGlu26−MIm complexes, respectively (see in the System Preparation section above). To relax the starting structures, both glycoside inhibitors were minimized by SD for 2000 steps, followed by CG for 1000 steps in the gas phase. The GAFF2 parameter was adopted to treat the inhibitor molecules. Each inhibitor was solvated by TIP3P water molecules in a 10 Å rectangular simulation box using periodic boundary conditions. The resulting systems were composed of 2078 atoms for the GIm system and 2123 atoms for the MIm system. To obtain the suitable number of REMD replicas for this explicit water system, a temperature predictor for REMD simulations was used [56,57], leading to 48 REMD replicas covering the range of temperatures from 300 to 500 K (see the exact replica temperatures for explicit water REMD simulations in Table S1). These conditions resulted in a good potential energy distribution overlap and temperature exchange acceptance ratio of around 60% (see SI Figure S2). Prior to performing REMD simulations, a short MD simulation of 5 ns was conducted for equilibrating the systems at each targeted temperature of each replica. Afterward, the REMD simulation was performed for 80 ns with a time step of 2 fs, in which the corresponding coordinates were collected every 2 ps (40,000 snapshots in total) from the REMD trajectories at 300 K for further conformational analysis of each system.

## 3. Results and Discussion

### 3.1. Structure of Rice Os3BGlu7 in Complex with Inhibitor

**Figure 2:**
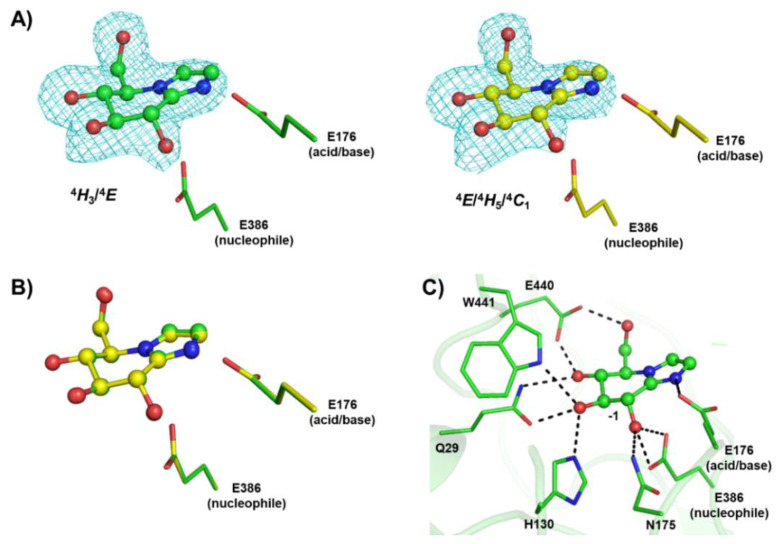
Structures of rice Os3BGlu7 in complex with glucoimidazole (GIm). (**A**) The Fo−Fc electron density omit maps of GIm are represented as a cyan mesh contoured at 3σ for molecules A (green) and B (yellow), (**B**) the superimposition of GIm in molecules A and B, and (**C**) hydrogen bonds between GIm and Os3BGlu7 at the −1 subsite are shown in black dashed lines.

Previously, we solved the structure of Os7BGlu26 β-mannosidase in complex with both GIm and MIm, each of which was found in the shape expected for their respective sugar TS conformation of ^4^*H*_3_/^4^*E* and *B*_2,5_ [29]. Initially, we soaked the Os3BGlu7 crystals with 50 mM MIm, as in that paper, but found GIm rather than MIm in the active site upon refining the structure. When a new batch of MIm that was carefully purified was soaked into crystals at 10 mM, GIm was still observed after refinement, while soaking at 1 mM MIm gave a weak GIm electron density in the active site. The data collection and structure refinement statistics for the final refined structure (PDB: 7BZM, soaked in 10 mM MIm) are shown in Table S2. This structure (refined at 2.3 Å resolution) clearly shows that the electron densities of ligand in the active sites of both protein molecules in the asymmetric unit fitted to the structure of GIm rather than the MIm (Figure 2A,B).

Since the Os3BGlu7 was reported to have a 4400-fold higher affinity (lower *K*_i_) for GIm in comparison to MIm [29], <0.1% of GIm contamination in the MIm preparation can logically explain this observation. Indeed, since the inhibition constant was determined with the same batch of MIm, a 0.022% contamination of GIm could account for the observed inhibition, if MIm did not contribute significantly to it. In this case, the MIm solution would have contained approximately 10 molecules of GIm per protein molecule in the crystal, which is more than enough to explain the observed density. GH1 enzymes are known to protonate the nitrogen of the imidazole ring [58]. This protonation could facilitate deprotonation of C2 and subsequent isomerization of MIm to GIm in the crystal, providing another possible explanation for the appearance of GIm in the active site.

The GIm was bound in the −1 subsite and formed hydrogen bonds with Os3BGlu7, including N175 and E386 at O2H, Q29, H130 and W441 at O3H, Q29 and E440 at O4H, E440 at O6H and E176 at N2 (Figure 2C). The GIm in molecule A showed a ^4^*E* conformation, consistent with the ^4^*H*_3_/^4^*E* TS proposed for hydrolysis of β-d-glucosides, while in molecule B the GIm showed a ring with Cremer–Pople parameters between ^4^*E*, ^4^*H*_5_ and ^4^*C*_1_ conformations, which is slightly distorted from the lowest energy ^4^*H*_3_ conformation [29]. The ^4^*E* conformation of GIm has previously been found in crystal structures of rice Os7BGlu26 β-glycosidase [29] and *Thermotoga maritima* (*Tm*GH1) β-glycosidase [59]. A standard relaxed ^4^*C*_1_ chair conformation was observed in the structures of rice Os3BGlu7 and its E176Q mutant in covalent complexes with 2-deoxy-2-fluoro-glucoside inhibitor [11,19] and a ^1^*S*_3_ screw boat conformation was detected in the −1 subsite of non-reducing glucosyl residue in the structures of Os3BGlu7 E176Q and E386G mutants with oligosaccharides [11,60]. The conformations of the GIm is consistent with the ^1^*S*_3_ → ^4^*E*/^4^*H*_3_^⧧^ → ^4^*C*_1_ conformational itinerary proposed for β-d-glucoside hydrolysis by Os3BGlu7 and other retaining β-glucosidases [11,15,59].

### 3.2. Conformational Dynamics of Protein—Inhibitor Complex

**Figure 3:**
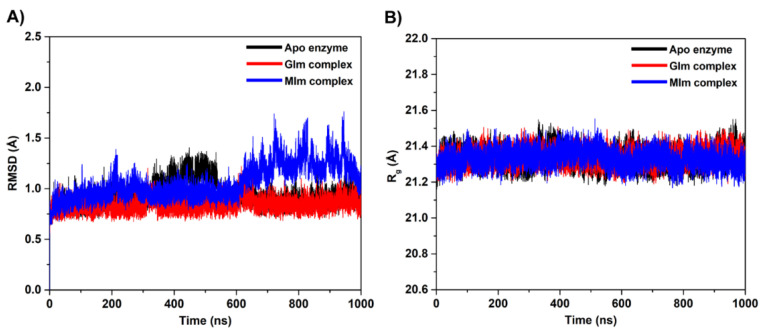
(**A**) Time evolution of the root-mean-square deviation (RMSD) of Cα atoms for each sytem. (**B**) Radius of gyration (R_g_) evolution for each system.

To estimate the quality of the MD simulations, the root-mean-square deviation (RMSD) with respect to the initial minimized structure for all Cα atoms of each system was calculated and plotted along the simulation time (Figure 3A). The RMSD profile of the GIm complex is found to behave similarly to the apo form with a fluctuation of ~0.7–1.0 Å, particularly for the last 400 ns. In contrast, the RMSD values observed for the MIm complex increase up to ~1.6 Å after 600 ns and then remain at a fluctuation of ~1.0–1.6 Å until approaching 1 μs. This finding suggests an increased flexibility of the complex form possibly at the enzyme-binding pocket, most likely owing to the presence of bound MIm molecule. To further support this observation, the RMSD calculation was performed based on the heavy atoms of binding site residues, i.e., residues within a 7-Å sphere of inhibitor. From the RMSD plots, it can be seen that the Os3BGlu7 binding site showed significant structural changes induced by MIm binding, especially from ~700 to 1000 ns with the high oscillation of RMSD up to ~3.0 Å, as opposed to the variable behavior of the apo state and the complex formation with GIm (see SI Figure S3A). Besides the 1 μs MD simulations, two additional 200 ns MD simulations of each complex system were carried out using the same starting structure but different initial velocities to evaluate the effect of statistical error on the simulation. The RMSD for each complex is rather consistent with the 1 μs simulation indicating a good quality of the simulations (Figure S4).

To get more information about the equilibrium conformation and the protein structure compactness, the radius of gyration (R_g_) was calculated over the course of the simulation. The obtained results display that the R_g_ values of all systems (Figure 3B) are ~21.3 Å, suggesting that the overall protein structure remains relatively compact during the simulation time. In addition, the R_g_ of amino acid residues within a 7-Å sphere of the inhibitor was calculated to explain the conformational flexibility of the protein-binding pocket caused by inhibitor binding. The R_g_ indicates a more compact structure of the apo protein and the GIm complex in comparison with the MIm complex (SI Figure S3B). This observation correlates well with the differences in the RMSD values of the MIm complex compared to the rest of the systems, reflecting the higher flexibility associated with MIm binding. Although there is slight structural variation found in the protein active site of the MIm system, analysis of the defined secondary structure of the protein (DSSP) framework [61] shows that the structural features of the protein are stable along the simulation time (Figure S5). Since our simulation model appeared stable, the trajectories extracted from 600 to 1000 ns of the 1 μs MD simulations were adopted as the production phase for further analysis.

### 3.3. Binding Affinity of Protein—Inhibitor Complex

To estimate the binding strength of both inhibitors toward rice Os3BGlu7 β-glucosidase, the MM/PB(GB)SA methods implemented in AMBER16 were applied. The binding free energies (Δ*G*_bind_) together with their corresponding energy components of the two complexes averaged over 4000 snapshots from the last 400 ns are shown in Table 1. Note that the entropic contribution (*T*Δ*S*) was calculated by a normal mode analysis [62] applied on only 200 snapshots, due to the high computational expense for the calculation.

For the interaction energy in gas phase (Δ*E*_MM_), the main contributor for the binding interaction between Os3BGlu7 and each inhibitor is the attractive electrostatic interactions (Δ*E*_ele_ in Table 1), which are larger than the van der Waals (Δ*E*_vdW_) energy by ~6-fold for the GIm complex and ~2-fold for the MIm complex. However, the strongly favorable electrostatic interactions are counteracted by the relatively greater unfavorable polar solvation free energy (${\Delta G}_{\mathrm{sol}}^{\mathrm{ele}}$) calculated with the PB or GB model, whereas the Δ*E*_vdW_ contribution and nonpolar solvation free energy (${\Delta G}_{\mathrm{sol}}^{\mathrm{nonpolar}}$) exhibit favorable contributions to the total binding free energy of both complexes. With a summation of the entropic contribution, both MM/PBSA and MM/GBSA methods agree with each other in predicting the binding affinities of the GIm complex (−9.62 and −20.18 kcal/mol) higher than that of the MIm complex (10.84 and −1.43 kcal/mol). The obtained results are in the same trend with the experimental binding free energy (Δ*G*_bind__(exp__)_) converted from *K*_i_ values of Os3BGlu7 in complex with the two inhibitors [29]. Nevertheless, the free energy of binding calculated with the MM/PB(GB)SA approaches still deviates significantly from the experimental values. This event was also commonly found in other protein—ligand complexes [63,64], as the prediction quality of these methods primarily depends on the length of simulation time, implicit solvent model, radii sets and sampling protocols [65]. To outperform the predicted binding energies for such complexes, a higher-level calculation using the QM/MM-GBSA method [66] was conducted, where the inhibitor molecule was treated as the QM region based on the SCC-DFTB semiempirical Hamiltonian. In our case, the result shows that the evaluation of the binding free energy calculated with the SCC-DFTB/MM-GBSA method is relatively improved, as compared to the conventional MM/PB(GB)SA free energy calculations, particularly for the MIm system. Based on the QM/MM-GBSA approach, the favorable contribution to the inhibitor bindings is a summation of the quantum mechanics (Δ*E*_QM_) and Δ*E*_vdW_ energies (Table 1). Similar to the MM/PB(GB)SA method, this large negative value is generally screened by the rather high positive value of the solvation free energy (Δ*G*_sol__(QM__-GBSA__)_), as referred to the dehydration penalty that involves the loss of solute—solvent interactions upon ligand binding to the protein [67,68]. Overall, a significant decrease in the magnitude of the binding preference of GIm to Os3BGlu7 is mainly due to the reduction in the interaction enthalpy (negative value) and solvation free energy (positive energy).

In addition, the binding free energies were calculated with the SIE method to further support the above conclusion. It can be clearly seen from the calculated results in Table S3 that the order of the binding free energy values correlates well with the experimentally derived data. Although the intermolecular coulomb interactions (Δ*E*_c_) is considered as the favorable force to the total binding interaction energy, this favorable contribution was commonly subdued by the unfavorable reaction energies (Δ*G*^R^). In contrast, the Δ*E*_vdW_ and molecular surface-based energies (γ·∆MSA) play a key role in the binding of the inhibitors. This result is strongly consistent with the MM/PB(GB)SA and QM/MM-GBSA calculations, as mentioned above.

To overcome the limitation of the implicit water model used in the MM/PB(GB)SA, QM/MM-GBSA and SIE methods, WaterSwap calculations that use explicit water were conducted. WaterSwap estimates the absolute binding free energy using a replica-exchange thermodynamic integration algorithm along a reaction coordinate that swaps the ligand of interest with an equivalent volume of explicit water molecules in the protein-binding pocket [51]. Five independent WaterSwap calculations were performed for each system starting from five different protein−ligand structures extracted from the last 400 ns of MD simulation in order to determine the robustness and reliability of the binding free energy estimation. WaterSwap simulations suggest that the average binding free energy of GIm to Os3BGlu7 β-glucosidase (−15.1 ± 0.4 kcal/mol) has the higher binding strength than Os3BGlu7−MIm complex (−10.6 ± 0.5 kcal/mol) (Table S4), which is in agreement with the experimental binding free energy in this case, although the calculated binding free energies slightly overestimate the true binding free energy. This may be because WaterSwap ignores some effects (e.g., the entropy cost of putting the ligand into the binding site) [51,52,53]. Altogether, five different binding free energy calculation approaches presented here successfully predict the binding affinity of the two Os3BGlu7–inhibitor complexes, in which the GIm molecule could inhibit this enzyme more efficiently than MIm, consistent with the magnitude of the experimental binding free energies.

### 3.4. Inhibitor Binding Pattern

**Figure 4:**
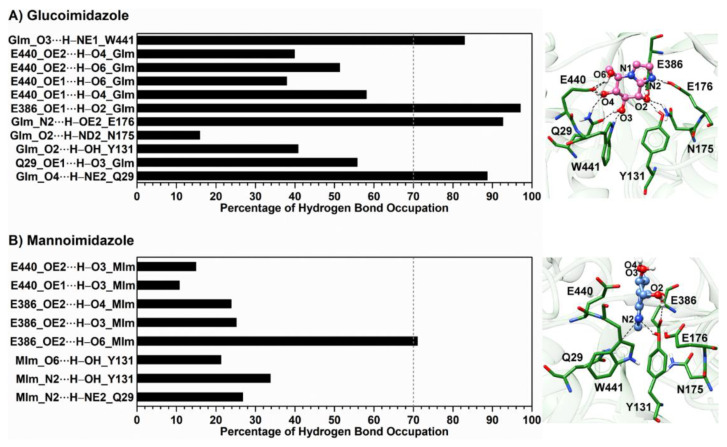
Percentage of hydrogen bond occupation together with the representative structures of the Os3BGlu7 residues accounting for (**A**) glucoimidazole and (**B**) mannoimidazole binding over the last 400 ns of MD simulations. Hydrogen bonds mainly formed between Os3BGlu7 residues and each inhibitor are represented by black dashed lines.

One of the crucial interactions involved in binding of the inhibitors is intermolecular hydrogen bonding with the targeted protein. To verify such interaction, the occupation of hydrogen bond pairs between each inhibitor and the surrounding residues was calculated, based on the two geometric criteria of (*i*) a proton donor (D) and acceptor (A) distance ≤ 3.5 Å, and (*ii*) a D–H·A bond angle ≥ 150° [69]. The results of hydrogen bond interactions and the representative structures illustrated from the last MD snapshot of the Os3BGlu7 β-glucosidase with each inhibitor bound are given in Figure 4, while the time evolution of these hydrogen bond distances is provided in Figure S6. The obtained results reveal that the GIm molecule forms several intermolecular hydrogen bonds with the polar/charged residues located in the Os3BGlu7 active site (Q29, Y131, N175, E176, E386, E440 and W441), as noted in the section describing the X-ray crystal structure. As compared to the X-ray structure of the related enzyme rice Os7BGlu26−GIm complex [29], the equivalent residues of rice Os3BGlu7 and Os7BGlu26 (Q29/Q32 and E440/E443) show a highly similar pattern of hydrogen bond interactions with GIm, as shown in Figure 2C. On the other hand, the MIm molecule forms hydrogen bonds with the smaller number of Os3BGlu7 residues (i.e., Q29, Y131, E386 and E440) and a rather low percentage of hydrogen bond occupation. It is noteworthy that the hydrogen bonds observed in the X-ray structure of the rice Os7BGlu26−MIm complex [29] are almost totally absent in the rice Os3BGlu7−MIm complex, since MIm is turned and twisted relative to the position of GIm, which binds similar to a substrate glycone [11]. This is probably due to lack of productive binding of MIm in the active site in Os3BGlu7, unlike Os7BGlu26, for which a crystal structure of the complex could be obtained. Furthermore, it is clearly seen that GIm forms stronger hydrogen bonds (>70% occupancy) than MIm. This leads to a significant decrease in MIm binding interactions by ~55 kcal/mol for electrostatic interactions calculated with the MM/PB(GB)SA methods, compared to the GIm complex (Table 1). Moreover, the lower binding efficiency of MIm toward Os3BGlu7 compared to that of GIm is possibly a result of the high structural flexibility of its sugar ring conformation in the enzyme-binding pocket, which points outwards from the binding site (see representative structure in Figure 4B). In contrast, the binding site of Os3BGlu7 β-glucosidase tends to accommodate the specific conformation of GIm well, resulting in the formation of more intermolecular hydrogen bonds and attractive interactions (discussed in more detail later).

**Figure 5:**
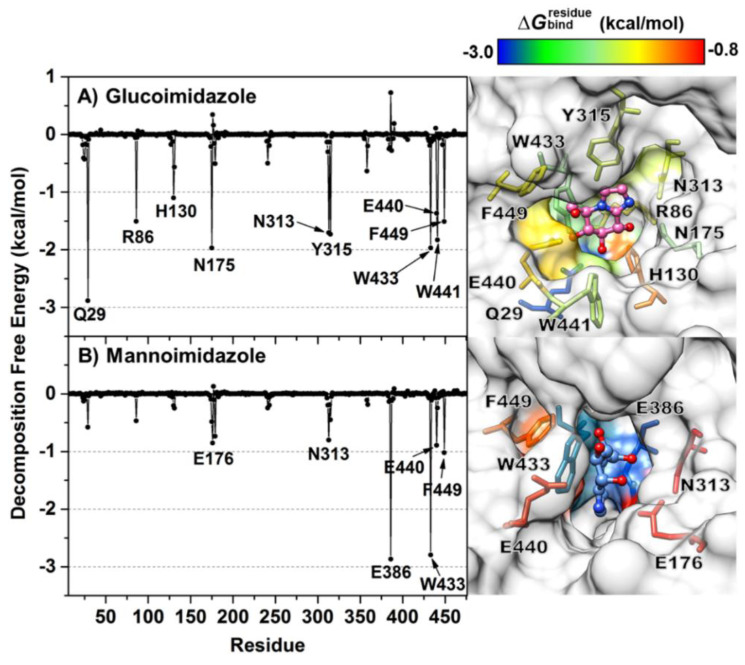
Per-residue decomposition free energy (kcal/mol) calculated with the MM/GBSA method for Os3BGlu7 in complex with (**A**) glucoimidazole and (**B**) mannoimidazole, where only residues involved in inhibitor binding (energy stabilization of <−0.8 kcal/mol) are colored on the basis of their ${\Delta G}_{\mathrm{bind}}^{\mathrm{residue}}$ values in the active site structures on the right. The residues with energy contribution ranging from −3.0 to −0.8 kcal/mol are shaded from blue to red, respectively. Note that the binding orientations of both complexes are drawn from the last MD snapshot of each system.

To clarify which amino acid residues contribute most to the inhibitor binding through the simulation period, the MM/GBSA residue-wise binding energy decompositions (${\Delta G}_{\mathrm{bind}}^{\mathrm{residue}}$) were analyzed and are illustrated in Figure 5. Note that the negative ${\Delta G}_{\mathrm{bind}}^{\mathrm{residue}}$ value indicates favorable residues to inhibitor bindings and vice versa for the positive value. Although the chemical structures of the two epimeric inhibitors are indeed similar (Figure 1B,C), they show greatly different binding positions in the enzyme active site. From the fingerprint in Figure 5, there are ten (Q29, R86, H130, N175, N313, Y315, W433, E440, W441 and F449) and six amino acid residues (E176, N313, E386, W433, E440 and F449) involved in the binding of GIm and MIm, respectively. In the case of GIm, Q29 exhibits the strongest binding interaction with GIm (${\Delta G}_{\mathrm{bind}}^{\mathrm{residue}}$ of −2.88 kcal/mol, blue). This residue of Os3BGlu7, which is conserved among all GH1 family members, plays a major role in making bidentate hydrogen bonds with O3 and O4 atoms of GIm (Figure 4A, right), keeping the suitable position of GIm inside the active site. Another residue important for GIm binding is the N175, which makes a direct hydrogen bond to O2 of GIm. Mutations of the equivalent residue in the GH1 family and other GH families led to significant changes in substrate specificity and catalytic efficiency [70,71]. Likewise, W441 interacts with the O3 of the GIm molecule through a strong hydrogen bond with the similar magnitude of binding interaction to GIm as the N175. In contrast to W441, W433 is involved in pi−pi interactions with the inhibitor moiety. However, the calculation reveals that the catalytic residue E386 exerts a moderate destabilizing effect on GIm binding (${\Delta G}_{\mathrm{bind}}^{\mathrm{residue}}$ of ~0.7 kcal/mol), possibly due to the repulsive electrostatic interaction between the negative charge of E386 and imidazole ring of GIm molecule.

On the other hand, interactions with the active site residues Q29, R86, H130, N175, Y315 and W441 are almost totally absent in the MIm complex, as the ${\Delta G}_{\mathrm{bind}}^{\mathrm{residue}}$ values of these residues are larger than −0.5 kcal/mol, suggesting that the Os3BGlu7 glycone-binding pocket does not prefer to bind to MIm. This is because of the high mobility of the MIm molecule inside the enzyme-binding pocket (discussed later), culminating in the loss of the main protein—inhibitor interactions. Instead, the E386 and W433 preferentially interact with the MIm through electrostatic attractions and pi−pi interactions, respectively. It can be noticed that even though Os3BGlu7 has the highest contribution with MIm binding directly via the catalytic nucleophile residue E386 (${\Delta G}_{\mathrm{bind}}^{\mathrm{residue}}$ of −2.87 kcal/mol, blue), it might not be strong enough to occupy in the −1 subsite of the enzyme active site, most likely owing to the lack of the other relevant interactions. This finding together with the intermolecular hydrogen bond analysis mentioned earlier support the experimental evidence for the weaker binding of MIm than GIm to Os3BGlu7 [29].

### 3.5. Ligand-Binding Pocket Volume and Water Accessibility in the Binding Pocket

As mentioned in the previous section, MIm is not fitted well in the Os3BGlu7 glycone-binding site. To determine such dynamics behavior, 400 representative structures over the time period of 600–1000 ns MD simulation were extracted every 1 ns to analyze the volume of ligand-binding pocket using ligand-based pocket scheme implemented in POcket Volume Measurer (POVME) 3.0 software [72]. The calculated pocket volume of the GIm complex is 272.3 ± 49.4 Å^3^, which is significantly lower than that of the MIm complex (330.4 ± 50.5 Å^3^), as shown in Figure S7. This relates to the higher binding pocket flexibility when the MIm molecule binds to the Os3BGlu7 active site. In other words, the ligand-binding pocket residues in the GIm system are more close-packed than in the MIm system, accommodating the tighter binding of GIm to Os3BGlu7.

The alteration in binding pocket volume induced by inhibitor binding could also make a change in the accessibility for water molecules to the Os3BGlu7 active-site pocket. Our assumption is that the system with a larger ligand-binding pocket should have more water molecules inside the enzyme-binding site, which could reasonably affect the protein−inhibitor interactions. To test this hypothesis, the radial distribution function (RDF) was used to investigate the distribution of water oxygen atoms around inhibitor heteroatoms (N1, O1, O2, O3, O4, O6 and N2; see Figure 1B,C for definition) in each complex.

**Figure 6:**
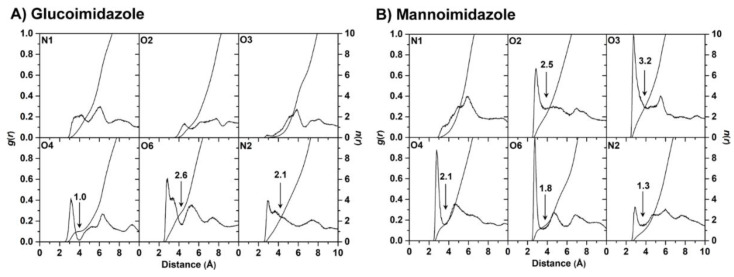
Radial distribution functions, *g*(*r*), of water oxygen atom and integration number, *n*(*r*), up to the first minimum around the heteroatoms (black arrow) of (**A**) glucoimidazole and (**B**) mannoimidazole in complex with the Os3BGlu7.

According to the RDF plots coupled with the integral of RDF values (*n*(*r*)) depicted in Figure 6, there is a broad peak detected within ~3.5 Å of the N1, O2 and O3 atoms in the GIm system, suggesting that the accessible water molecules weakly interact with these atoms. Meanwhile, the rest of heteroatoms show the first rather sharp peak centered at ~3 Å, accounting for a relatively high-water accessibility. The nonzero value at the first minimum observed in the RDF plots of the O6 and N2 atoms indicate a high level of water transfer in the first solvation shell. Conversely, the O4 atom is stably solvated by one water molecule, as can be seen by the first minimum at ~4 Å, approaching to zero value. In the case of the MIm complex, the first sharp peak of water distribution around MIm heteroatoms (except for the N1 atom) occurs at ~3 Å associated with a highly possible hydration interaction. Among all these atoms, a degree of transfer of water molecules is also high in the first shell of solvation. Taken together, more water molecules (approximately estimated from the total integration number) can solvate most of the heteroatoms of MIm in the active-site pocket relative to those of the GIm complex. The larger number of water molecules in the ligand-binding pocket of the MIm system tends to disrupt the direct protein—ligand interactions and consequently relates to the decrease in dehydration penalty (Table 1). This conclusion can be supported by the calculation of the number of hydrogen bonds between the inhibitor and water molecules over the course of the simulation (Figure S8A). The results reveal that the number of solute-solvent hydrogen bonds in the Os3BGlu7−GIm complex is evidently less than that in the Os3BGlu7−MIm complex, in which they probably replace the direct interactions between protein and inhibitor. However, such interacting water molecules are also able to form bridges of protein–water–ligand that may stabilize the complex, as seen in Os3BGlu7 interactions with oligosaccharides [11]. Thus, the number of such water bridging interactions between each inhibitor and active site residues was tracked during the simulation (Figure S8B). The calculation displays that the number of bridging water molecules is relatively low (~1–2 bridging hydrogen bonds for the GIm complex and ~3–4 for the MIm complex) with a maximum percentage of occupancy of only ~15% and ~9% for the respective GIm and MIm systems (data not shown), reflecting that water molecules occupying the ligand-binding cavity of Os3BGlu7 play a minor role in bridging between the protein and inhibitors. In summary, the binding of MIm to Os3BGlu7 results in the more accessible water molecules inside the binding pocket of Os3BGlu7 and thereby affects the direct protein—ligand interactions instead of making a bridging water network.

### 3.6. Sugar Ring Conformation

**Figure 7:**
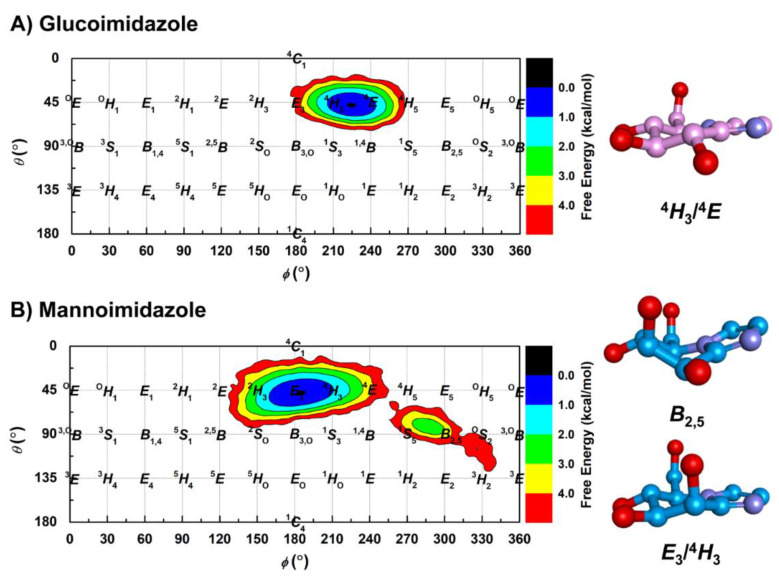
Conformational free energy landscapes of the Cremer—Pople parameters together with the representative sugar ring conformation of (**A**) glucoimidazole and (**B**) mannoimidazole bound to Os3BGlu7 along the 1-μs MD simulation.

The two epimeric sugar-shaped heterocyclic inhibitors presented here (GIm and MIm) are used for imitating the structural and electronic properties of the oxocarbenium ion-like TS during glycosidase catalysis [73,74]. Nonetheless, the degree of TS mimicry of these inhibitors upon molecular complexation with the enzyme is different. To explore the conformational preferences of the β-d-glucosyl and β-d-mannosyl moieties in the two Os3BGlu7−inhibitor complexes, the Cremer−Pople [33] puckering coordinates *θ* and *ϕ* were monitored along the MD simulation. All possible conformations of the β-d-glucosyl and β-d-mannosyl moieties on the respective GIm and MIm. The Cremer−Pople *θ* and *ϕ* puckering coordinates of the d-glucosyl moiety show the most populated distribution centered between the ^4^*H*_3_ and ^4^*E* conformations (Figure 7A). This finding correlates with its free form in solution derived from our REMD simulation, which exhibits a mixture of *E*_3_, ^4^*H*_3_ and ^4^*E* conformations (Figure S9A), corresponding to the energy minimum in its conformational free energy landscape [29]. This indicates that the d-glucosyl moiety remains close to its low energy conformation upon binding to Os3BGlu7. The ^4^*H*_3_/^4^*E* conformation was also observed in the X-ray structure of GIm in complex with Os3BGlu7 (Figure 2), as well as those of other GH1 enzymes [29,59,75]. This is consistent with β-d-glucoside hydrolysis by Os3BGlu7 proceeding via a ^1^*S*_3_ → ^4^*H*_3_/^4^*E*^⧧^ → ^4^*C*_1_ conformational itinerary, as previously proposed [11] and observed in the QM/MM metadynamics simulations of Os7BGlu26 β-glycosidase with β-d-glucoside substrate [29].

Likewise, the same calculations performed on MIm provide strikingly similar results as compared to GIm. The most populated distribution of the d-mannosyl moiety suggests that the conformation is located in a similar region of the Mercator diagram, between *E*_3_ and ^4^*H*_3_ (Figure 7B), although the starting conformation of the MIm d-mannosyl moiety obtained from the superimposition with the X-ray structure of Os7BGlu26−MIm complex is *B*_2,5_ conformation. The conversion of MIm from a *B*_2,5_ to a *E*_3_/^4^*H*_3_ conformation occurs at the beginning of the MD simulation (~20 ns) and it remains in that conformation until the end of the simulation time (Figure S10). This observation is strongly consistent with the results from our REMD simulation, in which the preferential conformations of the d-mannosyl group on the MIm molecule free in aqueous solution are centered at *E*_3_ and ^4^*H*_3_ conformations (Figure S9B), matching the lowest energy region of the conformational free energy landscape [29]. However, this dominant conformation suggested from our simulations contrasts the *B*_2,5_ conformation seen for MIm bound to rice GH1 β-glycosidase Os7BGlu26 [29], *B*. *thetaiotaomicron* GH2 β-mannosidase [76], and *C*. *perfringens* GH125 α-1,6-mannosidase [77]. This implies that the binding of MIm at the −1 subsite of Os3BGlu7 does not provide the energy needed to distort it toward the mannoside TS conformation seen in enzymes preferring mannoside hydrolysis. Consequently, it adopted its lower energy *E*_3_/^4^*H*_3_ sugar ring conformation, twisted, and moved farther out in this subsite (see representative structure in Figure 5B) to a position where MIm could bind more favorably in the Os3BGlu7 active site. Similarly, the binding preference of MIm with a ^4^*H*_3_ conformation at the +1 subsite was also reported in the X-ray structure of MIm bound to *C*. *perfringens* GH125 α-1,6-mannosidase with 100% occupancy, while a *B*_2,5_ conformation of the MIm molecule was found at the −1 subsite with approximately 70–80% occupancy [77]. Subsequently, it can be inferred that MIm does not achieve TS mimicry in the Os3BGlu7 active site, explaining its relatively low binding energy in comparison with GIm. Indeed, GIm exhibited tighter binding to three GH1 enzymes (Os3BGlu7, Os7BGlu26 and HvBII) than MIm, even though Os7BGlu26 and HvBII prefer mannoside substrates [29]. The energy required for distortion from the low energy *E*_3_/^4^*H*_3_ to the *B*_2,5_ TS conformation of mannosidases (approximately 6 kcal/mol) [29] appears to come at a cost of binding energy that is not overcome by interactions with the Os3BGlu7 active site. The *B*_2,5_ conformation is needed in order for mannose to make all the optimal sugar binding residues in the active site, similar to glucose in the ^4^*H*_3_/^4^*E* conformation in the catalytic position in the active site of Os7BGlu26. For Os3BGlu7, it appears that the binding of MIm in another position with fewer interactions, but without distortion, is more favorable than binding in the distorted shape in the catalytically productive position. If this is also true for mannoside substrates, it could explain its lower activity on mannoside substrates compared to the highly similar Os7BGlu26 and HvBII [78].

## 4. Conclusions

We have shown that GIm binds tightly in the −1 subsite of Os3BGlu7 in its relaxed ^4^*H*_3_/^4^*E* conformation, corresponding to the TS structure for β-glucosidases. In contrast, MIm is displaced by a slight contaminant of GIm in the crystal structure. Our MD simulations showed that, indeed, unlike the mannoside-preferring Os7BGlu26, which binds MIm through intermolecular interactions similar to GIm in the glycone-binding site, the glucoside-preferring Os3BGlu7 prefers to bind in a relaxed *E*_3_/^4^*H*_3_ conformation in the active site entrance. This suggests that the energetic cost of distorting MIm to the *B*_2,5_ conformation for optimal binding in the −1 subsite is not sufficiently compensated by the −1 subsite interactions, while binding with relaxed configuration in the active site entrance is more energetically favorable. If a similar logic can be applied to mannoside substrates, it may explain the relative substrate preferences of Os3BGlu7 for glucosides and Os7BGlu26 for mannosides, despite the high similarity of their active sites.

## Figures and Tables

**Table 1:** Binding free energies (kcal/mol) calculated with the Molecular Mechanics/Poisson–Boltzmann (generalized Born) Surface Area (MM/PB(GB)SA) and Quantum Mechanics/generalized Born Surface Area (QM/MM-GBSA) methods. Errors labeled by the signs ± represent the standard errors of mean (SEM).

Energy Component	GIm Complex	MIm Complex
Gas term		
Δ*E*_vdW_	−15.63 ± 0.06	−19.65 ± 0.05
Δ*E*_ele_	−89.43 ± 0.09	−34.05 ± 0.15
Δ*E*_MM_	−105.06 ± 0.07	−53.70 ± 0.15
Δ*E*_QM_	−79.02 ± 0.08	−30.78 ± 0.13
−*T*Δ*S*	11.30 ± 0.40	13.79 ± 0.32
**Solvation term**		
**PBSA**		
${\Delta G}_{\mathrm{sol}(\mathrm{PBSA})}^{\mathrm{ele}}$	86.43 ± 0.06	53.35 ± 0.14
${\Delta G}_{\mathrm{sol}(\mathrm{PBSA})}^{\mathrm{nonpolar}}$	−2.29 ± 0.00	−2.60 ± 0.00
Δ*G*_sol__(__PBSA__)_	84.14 ± 0.06	50.75 ± 0.14
GBSA		
${\Delta G}_{\mathrm{sol}(\mathrm{GBSA})}^{\mathrm{ele}}$	77.38 ± 0.06	42.05 ± 0.12
${\Delta G}_{\mathrm{sol}(\mathrm{GBSA})}^{\mathrm{nonpolar}}$	−3.80 ± 0.00	−3.56 ± 0.00
Δ*G*_sol__(__GBSA__)_	73.58 ± 0.06	38.48 ± 0.12
QM-GBSA		
${\Delta G}_{\mathrm{sol}(\mathrm{QM}\text{-}\mathrm{GBSA})}^{\mathrm{ele}}$	65.40 ± 0.05	34.35 ± 0.10
${\Delta G}_{\mathrm{sol}(\mathrm{QM}\text{-}\mathrm{GBSA})}^{\mathrm{nonpolar}}$	−3.80 ± 0.00	−3.56 ± 0.00
Δ*G*_sol__(__QM__-__GBSA__)_	61.60 ± 0.05	30.79 ± 0.10
**Binding free energy**		
Δ*G*_bind__(__MM__/__PBSA__)_	−9.62	10.84
Δ*G*_bind__(__MM__/__GBSA__)_	−20.18	−1.43
Δ*G*_bind__(__QM__/__MM__-__GBSA__)_	−21.75	−5.85
Δ*G*_bind__(__inhibition__)_^1^ [29]	−13.00	−7.90

^1^ Experimental binding free energy values, Δ*G*_bind__(exp__)_, were obtained using the relation of Δ*G* = *RT*ln*K*_i_ based on experimental competitive inhibition constant *K*_i_ at 303 K [29].

## Supplementary Materials

The following are available online at https://www.mdpi.com/2218-273X/10/6/907/s1, Figure S1: The initial conformation used for MD simulation, Table S1: REMD temperatures setting for the explicit solvation simulation, Table S2: X-ray data collection and structure refinement statistics for rice Os3BGlu7 with glucoimidazole structure, Figure S2: Potential energy distribution of each inhibitor, Figure S3: (A) Time evolution of the RMSD of the heavy atoms of residues in the active site. (B) Time evolution of the radius of gyration (R_g_) of the active site residues for each system, Figure S4: Time evolution of the RMSD of Cα atoms for two additional MD simualtions, Figure S5: DSSP plot of the secondary structure of Os3BGlu7 in complex with (A) glucoimidazole and (B) mannoimidazole, Table S3: Binding free energies (kcal/mol) calculated with the solvated interaction energy method, Table S4: Absolute binding affinity (kcal/mol) of Os3BGlu7 in complex with glucoimidazole and mannoimidazole calculated with the WaterSwap approach, Figure S6: Time evolution of the lengths of hydrogen bonds, Figure S7: Volume of ligand-binding pockets of the Os3BGlu7 in complex with glucoimidazole (red line) and mannoimidazole (blue line) over the last 400 ns of MD simulation, Figure S8: (A) Number of hydrogen bonds formed between each inhibitor and water molecules as well as (B) number of water bridging interactions along 1-μs MD simulation, Figure S9: Conformational free energy landscapes of the Cremer−Pople parameters, Figure S10: Time evolution of the Cremer—Pople parameters.
