# Supplementary material for: Structural Basis of Specific Glucoimidazole and Mannoimidazole Binding by Os3BGlu7
Source: Biomolecules. 2020 Jun 15;10(6):907. doi: 10.3390/biom10060907 (PMC7356692; doi:10.3390/biom10060907)
Supplement: Supplementary file 1 [file biomolecules-10-00907-s001.pdf]

Supplementary data

Table of Contents

Figure S1 2

Table S1 3

Table S2 4

Figure S2 5

Figure S3 6

Figure S4 7

Figure S5 8

Table S3 9

Table S4 10

Figure S6 11

Figure S7 12

Figure S8 13

Figure S9 14

Figure S10 15

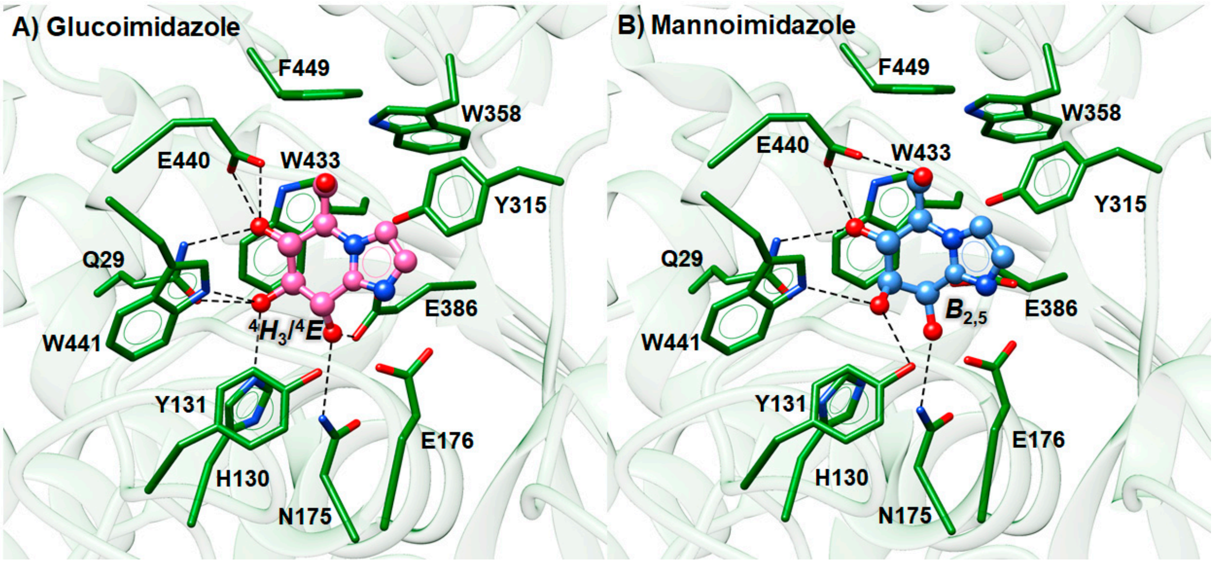

Figure S1: The initial conformation of (**A**) glucoimidazole and (**B**) mannoimidazole bound to the active site of Os3BGlu7 β-glucosidase used for MD simulation. Note that the black dashed line indicates hydrogen bond formation.

Table S1: REMD temperatures setting for the explicit solvation simulation.

#Replica	Temperature	#Replica	Temperature
1	300.00	25	389.91
2	303.29	26	394.18
3	306.61	27	398.51
4	309.97	28	402.87
5	313.37	29	407.29
6	316.81	30	411.75
7	320.28	31	416.27
8	323.80	32	420.80
9	327.35	33	425.41
10	330.94	34	430.07
11	334.57	35	434.78
12	338.24	36	439.54
13	341.95	37	444.36
14	345.82	38	449.23
15	349.62	39	454.15
16	353.45	40	459.12
17	357.33	41	464.15
18	361.25	42	469.24
19	365.21	43	474.38
20	369.22	44	479.57
21	373.27	45	484.69
22	377.36	46	490.00
23	381.50	47	495.36
24	385.68	48	500.00

Table S2: X-ray data collection and structure refinement statistics for rice Os3BGlu7 with glucoimidazole structure.

Data set (PDB ID)	Os3BGlu7 with glucoimidazole (7BZM)*
**Data collection**	
Space group	$P2_12_12_1$
Cell dimensions	
a, *b*, *c* (Å)	*a* = 80.4, *b* = 101.6, *c* = 127.5
α, β, γ (°)	90, 90, 90
Resolution (Å)	50-2.30 (2.34-2.30)
$CC_{1/2}$	0.998 (0.947)
R_{merge} (%)	21.8 (83.8)
I / σ^I	17.3 (6.6)
Completeness (%)	100 (99.9)
Redundancy	6.9 (7.3)
**Refinement**	
Resolution (Å)	50-2.30
No. reflections	44707
R_{work} / R_{free} (%)	17.7/21.7
**No. atoms**	
Protein	3812/3801
Carbohydrate subsite -1	14/14
Hetero	35
Water	480
B-factors (Å^2)	
Protein	30.0/30.7
Carbohydrate subsite -1	32.1/33.9
Hetero	43.3
Water	36.4
R.m.s deviations	
Bond lengths (Å)	0.008
Bond angles (°)	1.27
Ramachandran plot	
Residues in most favorable regions (%)	89.1
Residues in allowed regions (%)	10.9

*Each dataset comprises data from a single crystal. Values in parentheses are for highest-resolution shell.

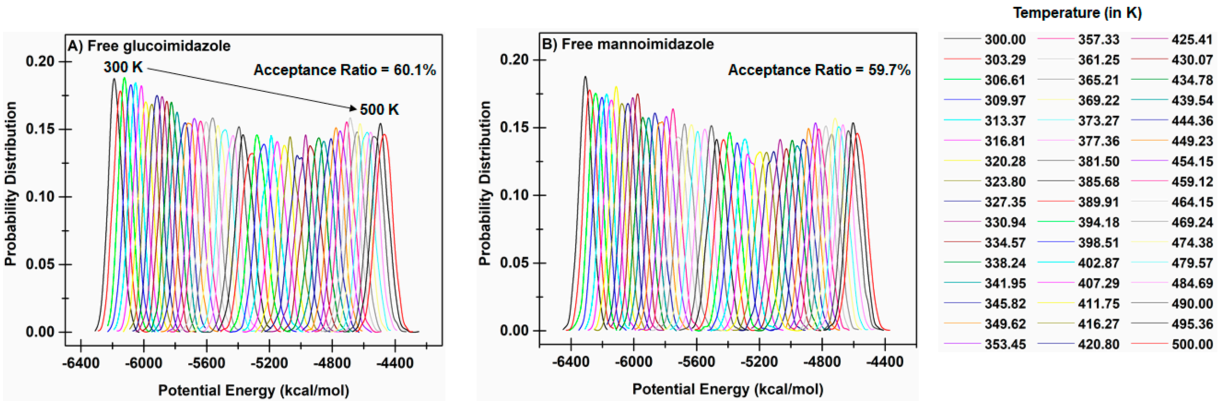

Figure S2: Potential energy distribution of each inhibitor for (**A**) free glucoimidazole and (**B**) free mannoimidazole during the production phase (80 ns) of REMD simulations, where the exchange acceptance ratio is also given.

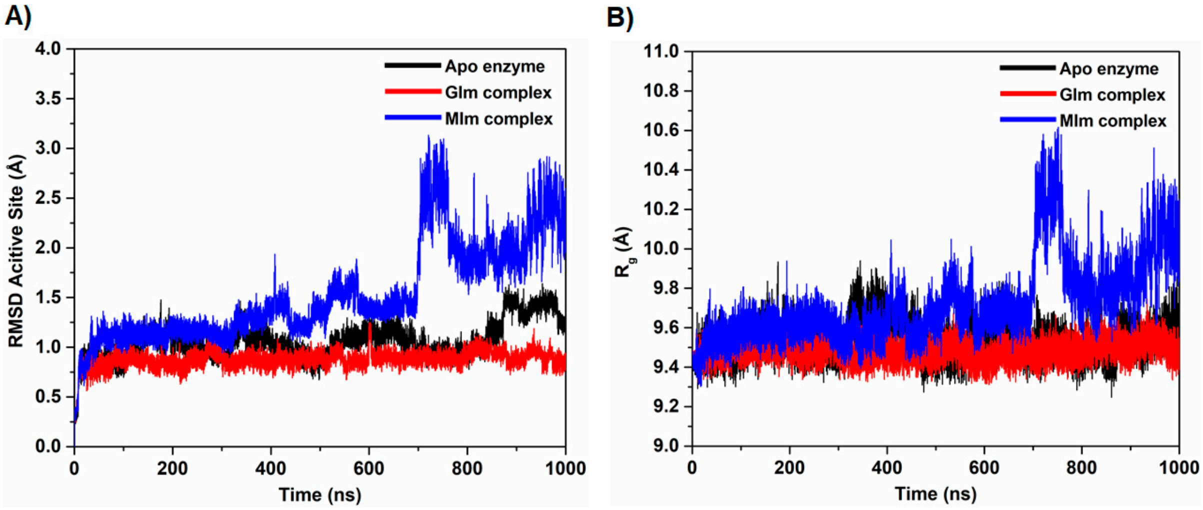

Figure S3: (**A**) Time evolution of the RMSD of the heavy atoms of residues in the active site (defined as amino acid residues within 7-Å sphere of inhibitor) for each system. (**B**) Time evolution of the radius of gyration (R_g) of the active site residues for each system.

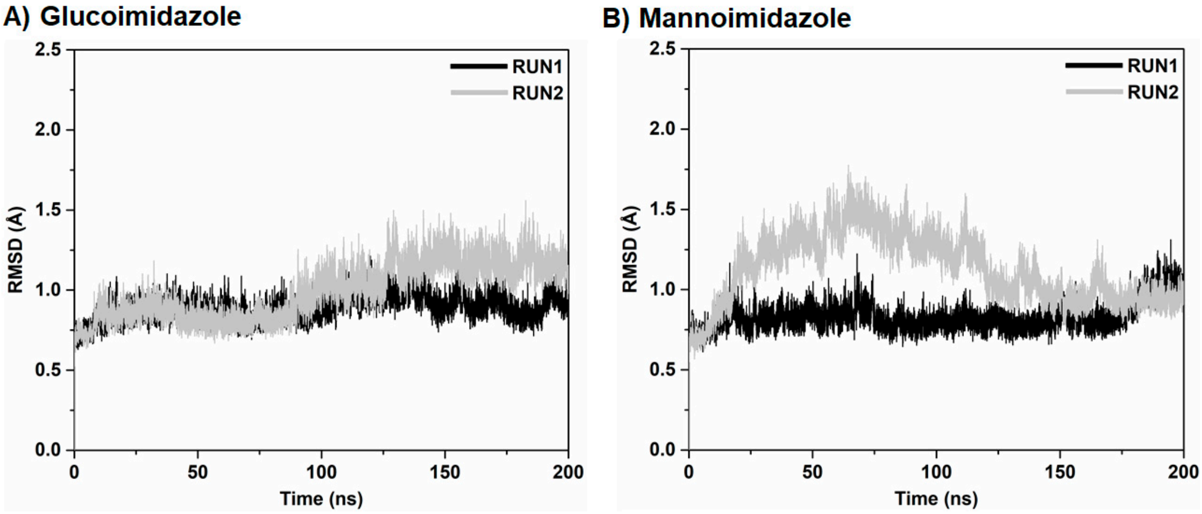

Figure S4: Time evolution of the RMSD of Cα atoms for two additional MD simualtions (defined as RUN1 and RUN2) for (**A**) Os3BGlu7−glucoimidazole and (**B**) Os3BGlu7−mannoimidazole complexes.

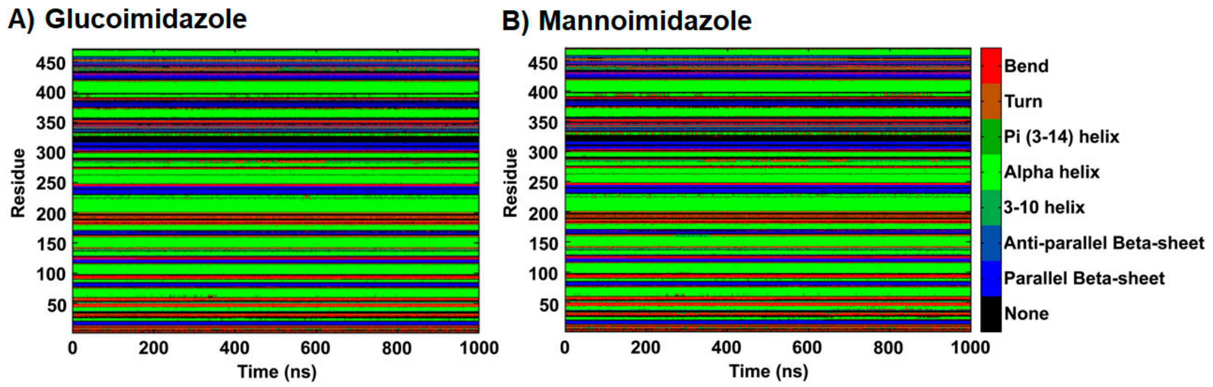

Figure S5: DSSP plot of the secondary structure of Os3BGlu7 in complex with (**A**) glucoimidazole and (**B**) mannoimidazole during MD simulation.

Table S3: Binding free energies (kcal/mol) calculated with the solvated interaction energy method. Errors labeled by the signs ± represent the SEM.

Energy component	GIm complex	MIm complex
ΔE_{vdW}	−15.63 ± 0.06	−19.65 ± 0.05
ΔE_{c}	−39.76 ± 0.04	−15.14 ± 0.07
$\gamma\cdot\Delta$MSA	−4.54 ± 0.01	−5.21 ± 0.01
ΔG^{R}	22.19 ± 0.02	14.37 ± 0.04
C	−2.89	
α	0.104758	
[a]ΔG_{bind}	−6.84	−5.57

[a]The binding free energies (ΔG_{bind}) computed by the SIE method were obtained from the following equation: $\Delta G_{\mathrm{bind}} = \alpha \times [E_{\mathrm{c}}(D_{\mathrm{in}}) + \Delta G^{\mathrm{R}} + E_{\mathrm{vdW}} + \gamma \cdot \Delta MSA(\rho)] + \mathrm{C}$, where E_{c} and E_{vdW} are the coulombic interaction and van der Waals interaction energies in the bound form, respectively. The term ΔG^{R} indicates the change of the reaction energy upon inhibitor binding and is calculated by solving the Poisson equation with the boundary element method [1]. The term $\gamma\cdot\Delta$MSA relates to the change of the molecular surface area induced by inhibitor binding. The solute interior dielectric constant (D_{in}), the molecular surface area coefficient (γ), the Amber van der Waals radii linear scaling coefficient (ρ), the global proportionality coefficient associated with the loss of conformational entropy upon binding (α) and a constant (C) are the parameters optimized by fitting to the absolute binding free energy for a set of 99 protein-ligand complex [2]. These optimized parameters are $D_{\mathrm{in}} = 2.25$, $\gamma = 0.0129$ kcal/(mol·Å^2), $\alpha = 0.104758$, and C = -2.89 kcal/mol, respectively.

Table S4: Absolute binding affinity (kcal/mol) of Os3BGlu7 in complex with glucoimidazole and mannoimidazole calculated with the WaterSwap approach. Alchemical transformations combined with RETI calculations were performed starting from 5 distinct snapshots selected from a classical MD trajectory for each complex system (see main text). Binding free energy values reported here are mean values ± the standard error of the mean (SEM).

	**Glucoimidazole**	**Mannoimidazole**
**Frame-01**	−14.5	−9.7
**Frame-02**	−15.4	−10.7
**Frame-03**	−15.4	−11.6
**Frame-04**	−14.1	−10.6
**Frame-05**	−16.1	−9.1
**Mean**	−15.1	−10.6
**SEM**	0.4	0.5

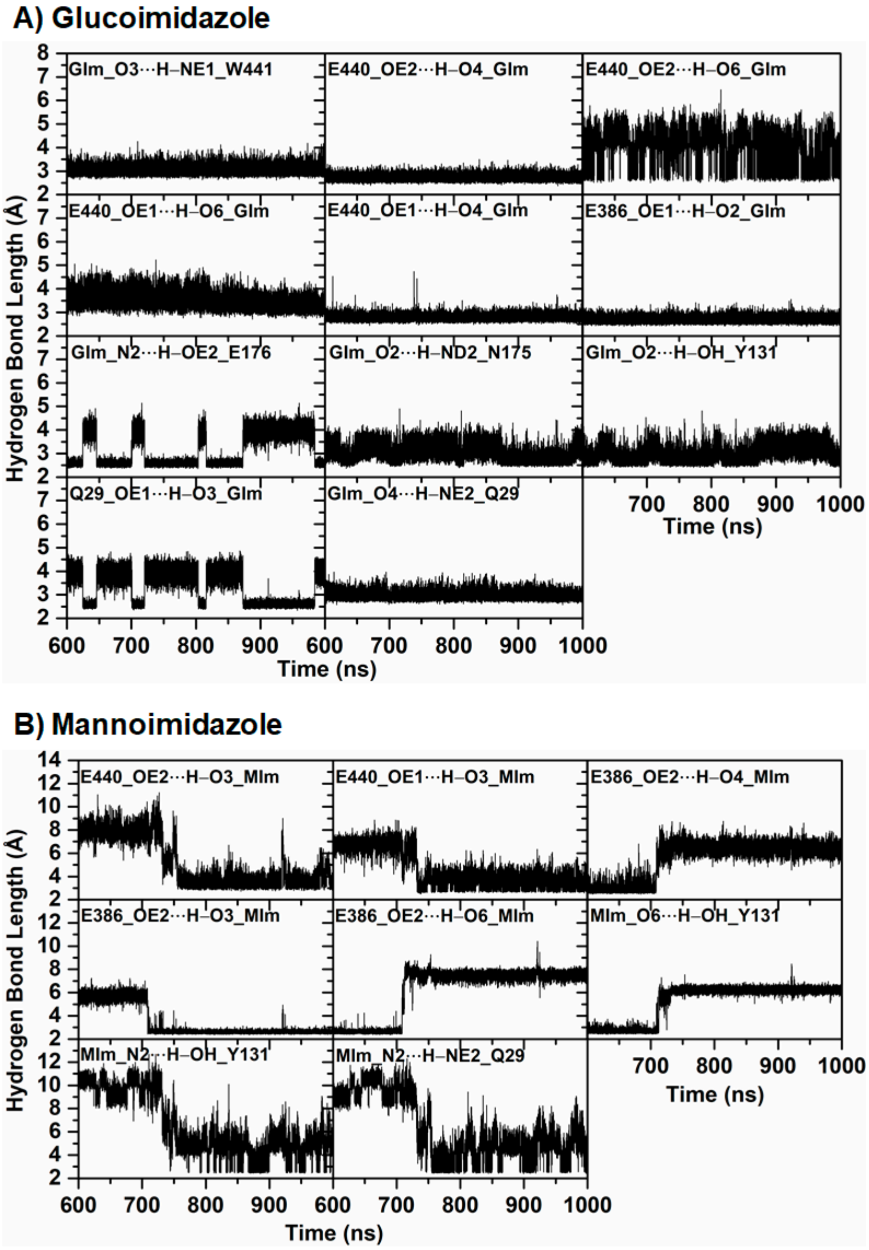

Figure S6: Time evolution of the lengths of hydrogen bonds shown in **Figure 4** of (**A**) glucoimidazole and (**B**) mannoimidazole bound to Os3BGlu7 over the last 400 ns of MD simulation.

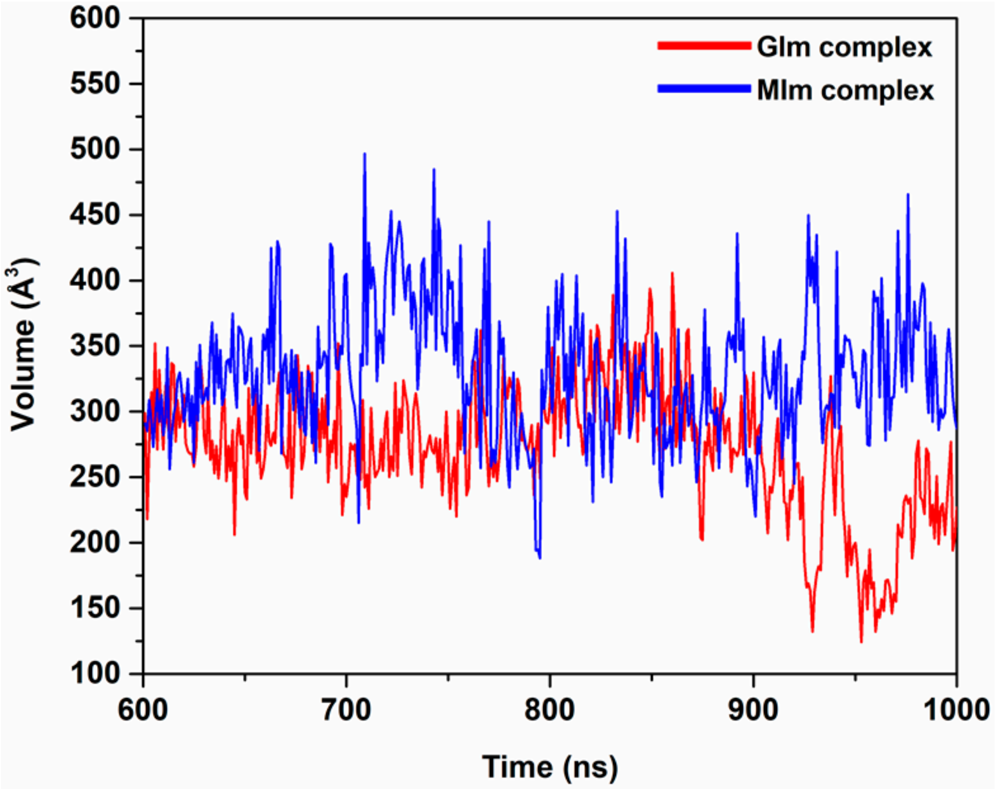

Figure S7: Volume of ligand-binding pockets of the Os3BGlu7 in complex with glucoimidazole (red line) and mannoimidazole (blue line) over the last 400 ns of MD simulation.

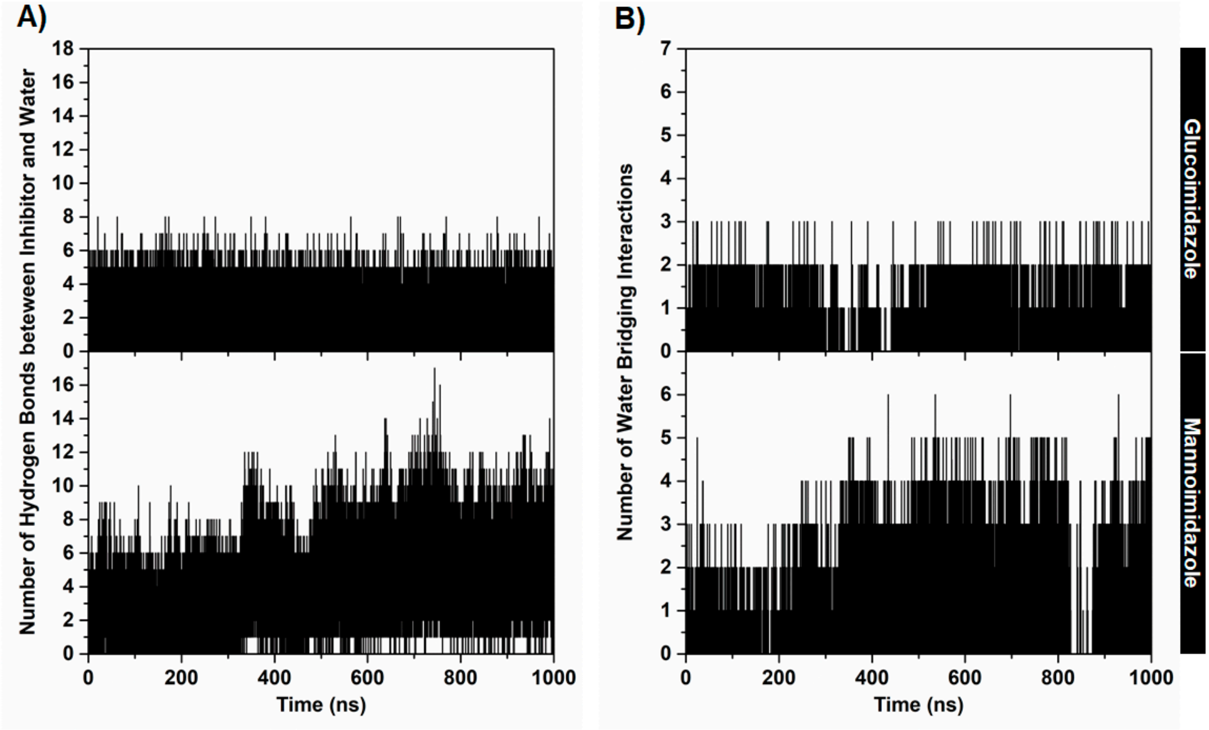

Figure S8: (**A**) Number of hydrogen bonds formed between each inhibitor and water molecules as well as (**B**) number of water bridging interactions along 1-μs MD simulation for (**top**) Os3BGlu7−glucoimidazole and (**bottom**) Os3BGlu7−mannoimidazole complexes.

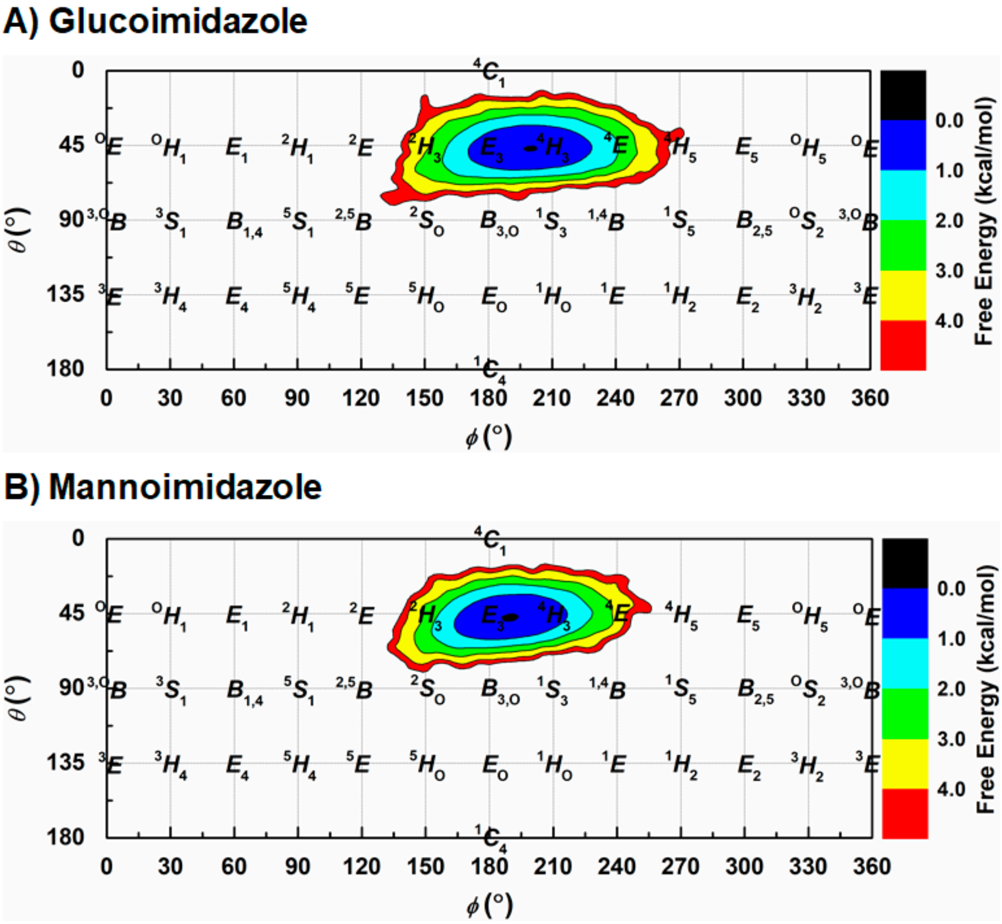

Figure S9: Conformational free energy landscapes of the Cremer−Pople parameters of (**A**) glucoimidazole and (**B**) mannoimidazole in the free form obtained from explicit solvent REMD simulations at 300 K.

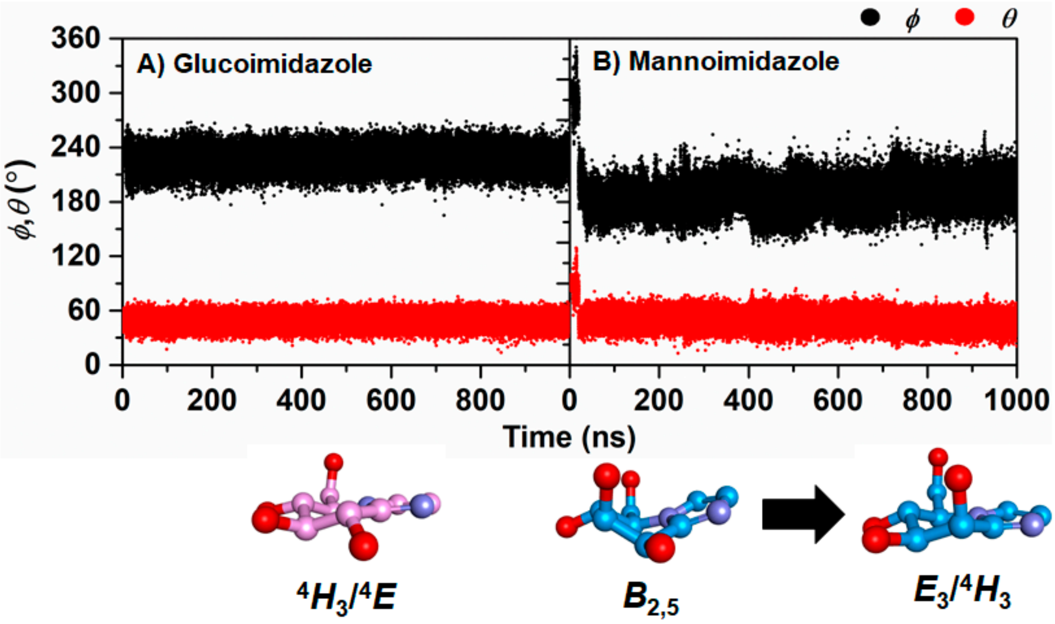

Figure S10: Time evolution of the Cremer−Pople parameters of (**A**) glucoimidazole and (**B**) mannoimidazole bound to Os3BGlu7 during MD simulation.

References

1. Purisima, E.O., *Fast summation boundary element method for calculating solvation free energies of macromolecules.* Journal of Computational Chemistry, 1998. **19**(13): p. 1494-1504.
2. Naïm, M., et al., *Solvated Interaction Energy (SIE) for scoring protein-ligand binding affinities. 1. Exploring the parameter space.* Journal of Chemical Information and Modeling, 2007. **47**(1): p. 122-133.
